# Genome wide analysis of MADS-box gene family in *Brassica oleracea* reveals conservation and variation in flower development

**DOI:** 10.1186/s12870-019-1717-y

**Published:** 2019-03-19

**Authors:** Xiao-Guang Sheng, Zhen-Qing Zhao, Jian-Sheng Wang, Hui-Fang Yu, Yu-Sen Shen, Xiao-Yuan Zeng, Hong-Hui Gu

**Affiliations:** 1 Institute of Vegetables, Zhejiang Academy of Agricultural Sciences, Hangzhou, 310021 China; 2 Agricultural Technology Promotion Station of Taizhou, Taizhou, 318000 China

**Keywords:** MADS-box genes, Phylogenetic analysis, Flower development, Expression patterns, *Brassica oleracea*

## Abstract

**Background:**

MADS-box genes play important roles in vegetative growth and reproductive development and are essential for the correct development of plants (particularly inflorescences, flowers, and fruits). However, this gene family has not been identified nor their functions analyzed in *Brassica oleracea*.

**Results:**

In this study, we performed a whole-genome survey of the complete set of MADS-box genes in *B. oleracea*. In total, 91 MADS-box transcription factors (TFs) were identified and categorized as type I (Mα, Mβ, Mγ) and type II (MIKC^C^, MIKC*) groups according to the phylogeny and gene structure analysis. Among these genes, 59 were randomly distributed on 9 chromosomes, while the other 23 were assigned to 19 scaffolds and 9 genes from NCBI had no location information. Both RNA-sequencing and quantitative real-time-PCR analysis suggested that MIKC genes had more active and complex expression patterns than M type genes and most type II genes showed high flowering-related expression profiles. Additional quantitative real-time-PCR analysis of pedicel and four flower whorls revealed that the structure of the *B.oleracea* MIKC genes was conserved, but their homologues showed variable expression patterns compared to those in *Arabidopsis thaliana*.

**Conclusion:**

This paper gives a detailed overview of the *BolMADS* genes and their expression patterns. The results obtained in this study provide useful information for understanding the molecular regulation of flower development and further functional characterization of MADS-box genes in *B. oleracea*.

**Electronic supplementary material:**

The online version of this article (10.1186/s12870-019-1717-y) contains supplementary material, which is available to authorized users.

## Background

The MADS-box family consists of genes encoding a class of transcription factors characterized by the presence of 58–60 conserved amino acids known as the MADS domain [1]. The MADS domain was named after the first four members of this family encoded by genes, *MINICHROMOSOME MAINTENANCE 1* (*MCM1*) from yeast [2], *AGAMOUS* (*AG*) from *Arabidopsis thaliana* [3], *DEFICIENS* (*DEF*) from *Antirrhinum majus* [4], and *SERUM RESPONSE FACTOR* (*SRF*) from *Homo sapiens* [5]. MADS-box genes are widely distributed in eukaryotes and play important roles in an organism’s growth and development, particularly in floral organ differentiation, flowering time regulation, and fruit development and ripening in angiosperms [6, 7].

Based on phylogenetic analysis, the MADS-box genes are divided into two categories: type I and type II [8]. All type II members in yeast, animal and plant are MEF2-like genes, while type I MADS-box genes in plant are SRF-like [8]. In addition to their common MADS (M) domain, MIKC genes also include intervening (I), keratin-like (K), and C-terminal (C) domains [9, 10]. M is the most conserved domain and located in the N-terminal region, which binds specific DNA through the CArG-box (5′-CC(A/T) 6GG-3′) in the promotor region of the target gene. I is a less conserved region consisting of approximately 30 amino acids and determines the specific binding of transcription factors to DNA and dimer formation. The K domain is the second conserved region forming a coiled-coil structure, which facilitates interactions between proteins. The C-terminal domain is the least conserved region, consists mainly of hydrophobic amino acids, and plays an important role in the formation and transcriptional activation of protein complexes [6, 11, 12]. Based on sequence and structure differences of the I domain, the MIKC genes are further divided into MIKC^C^ - and MIKC^∗^-types. MIKC^C^-type genes are the most widely studied MADS-box genes because they are essential in plant growth and development [13, 14]. According to the phylogenetic analysis of the M domain, type I genes (also known as M type genes) in plant are divided into four groups, Mа, Mß, Mγ, and Mδ. In *Arabidopsis*, Mδ genes are also assigned as MIKC^∗^-type genes based on their relatively close relationships with MIKC^C^-type genes [15].

Plant MIKC^C^ genes were demonstrated to play important roles in flower organogenesis [15]. Coen and Meyerowitz first proposed the famous ABC model, and later the integral ABCDE model was developed [16–18]. The identity of each whorl of floral organs was shown to be determined by different combinations of A, B, C, D and E genes activities: sepals (A + E), petals (A + B + E), stamens (B + C + E), carpels (C + E), and ovules (C + D + E) [17]. In *Arabidopsis*, functional genes were included in this model: *APETALA1* (*AP1*) is expressed in sepals and petals and belongs to the class A [19, 20]; *PISTILLATA* (PI) and *AP3* are expressed in petals and stamens and belong to the class B [21]; class C gene *AGAMOUS* (*AG*) is expressed in stamens and carpels [4, 22]; class D gene *AGAMOUS-LIKE 11*(*AGL11*, another name is *SEEDSTICK*, *STK*) is expressed in ovules [23]; class E genes *SEPALLATA 1* (*SEP1*), SEP2, SEP3 and SEP4, are expressed in all floral whorls [24, 25]. Other MIKC genes have been shown to possess different biological functions, such as genes regulating flowering time (*SOC1*, *SVP*, *FLC*, *AGL15*/*18*) [26–30]; fruit ripening genes (*SHP1*, *SHP2*, *FUL*) [31, 32]; and seed pigmentation genes (*TT16*) and root development genes (*AGL12*, *AGL17*) [33, 34].

*Brassica oleracea* contains multiple vegetable crops including broccoli, cauliflower, brussels sprouts, various types of cabbage, kohlrabi, and kale. Cauliflower is an economically important vegetable planted worldwide [35]. Compared with other crops of *B. oleracea*, cauliflower has a unique characteristic - a tight white flower curd, consisting of a large amount of shortened branches of indeterminate inflorescences [36]. Previous studies showed that the formation of cauliflower curd could be related to two MADS-box genes *BoCAL* and *BoAP1*, and in cauliflower the *bocal* mutant allele is present [37, 38]. However, other studies found only a weak connection between cauliflower phenotype and *boap1* and *bocal* mutations [39, 40]. Moreover, Lan and Paterson reported that curd-related traits are affected by 86 quantitative trait loci (QTLs), indicating that the cauliflower arrest is multi-gene controlled [41]. Genome-wide studies of MADS-box genes can be conducted, as the *B. oleracea* genome has been sequenced [42]. In this study, a total of 91 MADS-box genes were identified in the *B. oleracea* genome. The phylogenetic relationships, gene structure, chromosomal locations, and conserved motifs of the encoded proteins were analyzed. The tissue-specific expression of all *BolMADS* genes in cauliflower were further studied. This work provides useful information regarding the molecular mechanisms underlying cauliflower curd formation and flower development.

## Results

### Identification of MADS-box genes in *B. oleracea*

A total of 95 candidate genes were identified. Based on sequence analysis, four candidate MADS-box genes composed of approximately 200 bases were confirmed to contain incomplete MADS-box domains and then were excluded from the analysis. Therefore, a total of 91 MADS-box genes with complete and functional structures were identified in the *B. oleracea* genome. Among them, 82 genes were found in the *B. oleracea* genome database (named *BolMADS1*- *BolMADS82*), and 9 genes were acquired from the NCBI database (named with NCBI access numbers).

The MADS-box genes in *B. oleracea* genome had coding sequence lengths of 249–1221 base pairs. The encoded proteins length ranged from 82 to 406 amino acids with predicted molecular masses of 9.52–46.31 KDa and protein isoelectric points of 4.08–10.53 (Table 1).

**Table 1:** MADS-box gene family identified in *Brassica oleracea*

Gene name	Gene locus	Chr. no.	Protein		No. of introns	pI	Group
			Length/aa	Mol.wt./Kda			
*BolMADS1* (*BoFUL-a*)	*Bol007763*	Scaffold256	241	27.41	7	9.36	MIKC^c^
*BolMADS2* (*BoFUL-b*)	*Bol017059*	C07	241	27.52	7	9.46	MIKC^c^
*BolMADS3* (*BoFUL-c*)	*Bol036265*	C09	241	27.43	7	9.31	MIKC^c^
*BolMADS4*	*Bol029887*	C03	247	28.14	7	9.62	MIKC^c^
*BolMADS5*	*Bol030482*	C09	253	28.83	6	8.56	MIKC^c^
*BolMADS6*	*Bol041762*	C07	252	29.29	7	7.71	MIKC^c^
*BolMADS7*	*Bol011058*	C04	259	29.14	7	5.49	MIKC^c^
*BolMADS8*	*Bol024219*	Scaffold93_P1	240	27.42	6	7.14	MIKC^c^
*BolMADS9*	*Bol020052*	C02	160	18.69	3	9.62	MIKC^c^
*BolMADS10* (*BoCAL*)	*Bol024957*	C03	218	25.72	6	9.33	MIKC^c^
*BolMADS11*	*Bol034251*	C03	263	29.87	7	8.90	MIKC^c^
*BolMADS12*	*Bol029555*	C03	160	18.61	4	9.56	MIKC^c^
*BolMADS13*	*Bol023763*	C06	248	28.41	6	9.11	MIKC^c^
*BolMADS14*	*Bol004959*	C04	248	28.35	6	8.77	MIKC^c^
*BolMADS15*	*Bol037657*	Scaffold24	221	24.97	7	6.78	MIKC^c^
*BolMADS16*	*Bol033138*	C02	89	10.37	0	10.53	MIKC^c^
*BolMADS17*	*Bol036252*	C09	245	29.04	5	6.57	MIKC^c^
*BolMADS18*	*Bol017090*	C07	251	29.59	5	7.33	MIKC^c^
*BolMADS19*	*Bol025193*	C08	232	27.27	6	8.71	MIKC^c^
*BolMADS20*	*Bol032290*	C09	248	28.09	7	9.22	MIKC^c^
*BolMADS21*	*Bol041644*	Scaffold9_P1	224	26.51	6	7.85	MIKC^c^
*BolMADS22*	*Bol008758*	C03	216	24.28	5	9.10	MIKC^c^
*BolMADS23*	*Bol043693*	C09	130	14.54	2	9.57	MIKC^c^
*BolMADS24*	*Bol028071*	C03	202	23.16	6	9.36	MIKC^c^
*BolMADS25*	*Bol003519*	Scaffold348	212	24.03	6	6.35	MIKCc
*BolMADS26*	*Bol034952*	C02	211	23.90	6	7.66	MIKC^c^
*BolMADS27*	*Bol002366*	Scaffold392	211	23.97	6	6.85	MIKC^c^
*XP_013590290.1* ^a^ (*BoAP1-c*)	*XP_013590290.1*	**/**	256	30.13	7	8.31	MIKC^c^
*XP_013589842.1* (*BoAP1-a*)	*XP_013589842.1*	**/**	256	30.18	7	8.00	MIKC^c^
*XP_013600280.1*	*XP_013600280.1*	**/**	241	27.20	8	6.07	MIKC^c^
*XP_013631060.1*	*XP_013631060.1*	**/**	241	27.25	8	6.08	MIKC^c^
*XP_013597287.1*	*XP_013597287.1*	**/**	252	28.79	8	9.81	MIKC^c^
*XP_013626307.1*	*XP_013626307.1*	**/**	208	24.09	5	9.41	MIKC^c^
*XP_013607408.1*	*XP_013607408.1*	**/**	208	24.15	5	9.16	MIKC^c^
*XP_013630326.1*	*XP_013630326.1*	**/**	213	24.42	7	9.48	MIKC^c^
*XP_013632159.1*	*XP_013632159.1*	**/**	213	24.37	7	9.82	MIKC^c^
*BolMADS28*	*Bol042006*	Scaffold8_P1	335	38.03	9	4.95	MIKC^*^
*BolMADS29*	*Bol027523*	C06	329	37.65	9	5.03	MIKC^*^
*BolMADS30*	*Bol013125*	C01	207	23.94	6	9.56	MIKC^*^
*BolMADS31*	*Bol013192*	C08	338	38.88	10	6.09	MIKC^*^
*BolMADS32*	*Bol007216*	C07	389	44.21	10	6.86	MIKC^*^
*BolMADS33*	*Bol023922*	C06	406	46.31	8	5.80	MIKC^*^
*BolMADS34*	*Bol008953*	C02	257	29.09	1	9.28	Mα
*BolMADS35*	*Bol026349*	C06	213	24.22	0	5.48	Mα
*BolMADS36*	*Bol037864*	C04	187	21.32	0	5.07	Mα
*BolMADS37*	*Bol008951*	C02	123	14.29	0	9.90	Mα
*BolMADS38*	*Bol008950*	C02	120	13.98	0	9.95	Mα
*BolMADS39*	*Bol009782*	C08	206	23.45	0	9.37	Mα
*BolMADS40*	*Bol031566*	C08	162	18.43	0	8.74	Mα
*BolMADS41*	*Bol001956*	Scaffold412	176	20.03	0	6.98	Mα
*BolMADS42*	*Bol033229*	C04	240	27.83	1	9.47	Mα
*BolMADS43*	*Bol022502*	C05	171	19.07	0	5.4	Mα
*BolMADS44*	*Bol019111*	Scaffold133	189	21.44	0	9.15	Mα
*BolMADS45*	*Bol044870*	C06	256	28.53	0	4.43	Mα
*BolMADS46*	*Bol015619*	C06	247	27.56	0	4.40	Mα
*BolMADS47*	*Bol001334*	Scaffold454	193	22.36	0	9.03	Mα
*BolMADS48*	*Bol023453*	C08	268	29.59	0	4.76	Mα
*BolMADS49*	*Bol023443*	C08	268	29.58	0	4.76	Mα
*BolMADS50*	*Bol023442*	C08	268	29.57	0	4.76	Mα
*BolMADS51*	*Bol002451*	Scaffold388	364	40.07	0	4.51	Mα
*BolMADS52*	*Bol014335*	C03	198	21.87	0	4.93	Mα
*BolMADS53*	*Bol014328*	C03	247	27.44	0	5.17	Mα
*BolMADS54*	*Bol001745*	Scaffold424	263	29.06	0	4.08	Mα
*BolMADS55*	*Bol016615*	Scaffold153	325	36.41	0	4.28	Mα
*BolMADS56*	*Bol016587*	Scaffold153	285	32.00	0	4.97	Mα
*BolMADS57*	*Bol009950*	C04	359	39.83	0	4.39	Mα
*BolMADS58*	*Bol009965*	C04	159	17.76	0	6.12	Mα
*BolMADS59*	*Bol030956*	C01	344	39.50	0	8.33	Mβ
*BolMADS60*	*Bol016846*	C09	301	35.04	0	8.66	Mβ
*BolMADS61*	*Bol022880*	C03	127	14.73	0	8.42	Mβ
*BolMADS62*	*Bol002990*	Scaffold367	356	40.68	0	8.83	Mβ
*BolMADS63*	*Bol027893*	C03	274	31.42	0	8.44	Mβ
*BolMADS64*	*Bol008049*	C02	342	39.28	0	9.46	Mβ
*BolMADS65*	*Bol012873*	Scaffold192	324	37.21	0	5.69	Mβ
*BolMADS66*	*Bol012871*	Scaffold192	305	35.22	0	6.45	Mβ
*BolMADS67*	*Bol037792*	C04	82	9.52	1	5.23	Mβ
*BolMADS68*	*Bol044867*	C06	250	28.54	0	6.96	Mβ
*BolMADS69*	*Bol012124*	C04	178	20.55	0	9.58	Mβ
*BolMADS70*	*Bol010353*	C05	345	39.35	0	7.14	Mγ
*BolMADS71*	*Bol012341*	C07	157	18.42	0	9.85	Mγ
*BolMADS72*	*Bol012715*	Scaffold194_P1	395	44.69	0	5.72	Mγ
*BolMADS73*	*Bol012716*	Scaffold194_P1	395	44.64	0	5.54	Mγ
*BolMADS74*	*Bol012712*	Scaffold194_P1	395	44.72	0	5.61	Mγ
*BolMADS75*	*Bol023555*	Scaffold99_P1	162	18.89	0	9.8	Mγ
*BolMADS76*	*Bol002873*	Scaffold372	334	36.96	0	8.3	Mγ
*BolMADS77*	*Bol022282*	C07	375	42.74	0	5.25	Mγ
*BolMADS78*	*Bol004579*	C02	308	35.23	1	8.73	Mγ
*BolMADS79*	*Bol024509*	C02	252	28.89	0	5.43	Mγ
*BolMADS80*	*Bol002483*	Scaffold387	344	39.14	1	5.65	Mγ
*BolMADS81*	*Bol043965*	C09	191	21.98	0	5.05	Mγ
*BolMADS82*	*Bol036538*	C01	278	32.27	1	4.95	Mγ

^a^The genes of XP series were found in NCBI database, and there was no location information of *B. oleracea* genome

### Phylogenetic analysis of MADS-box gene family

**Fig. 1:**
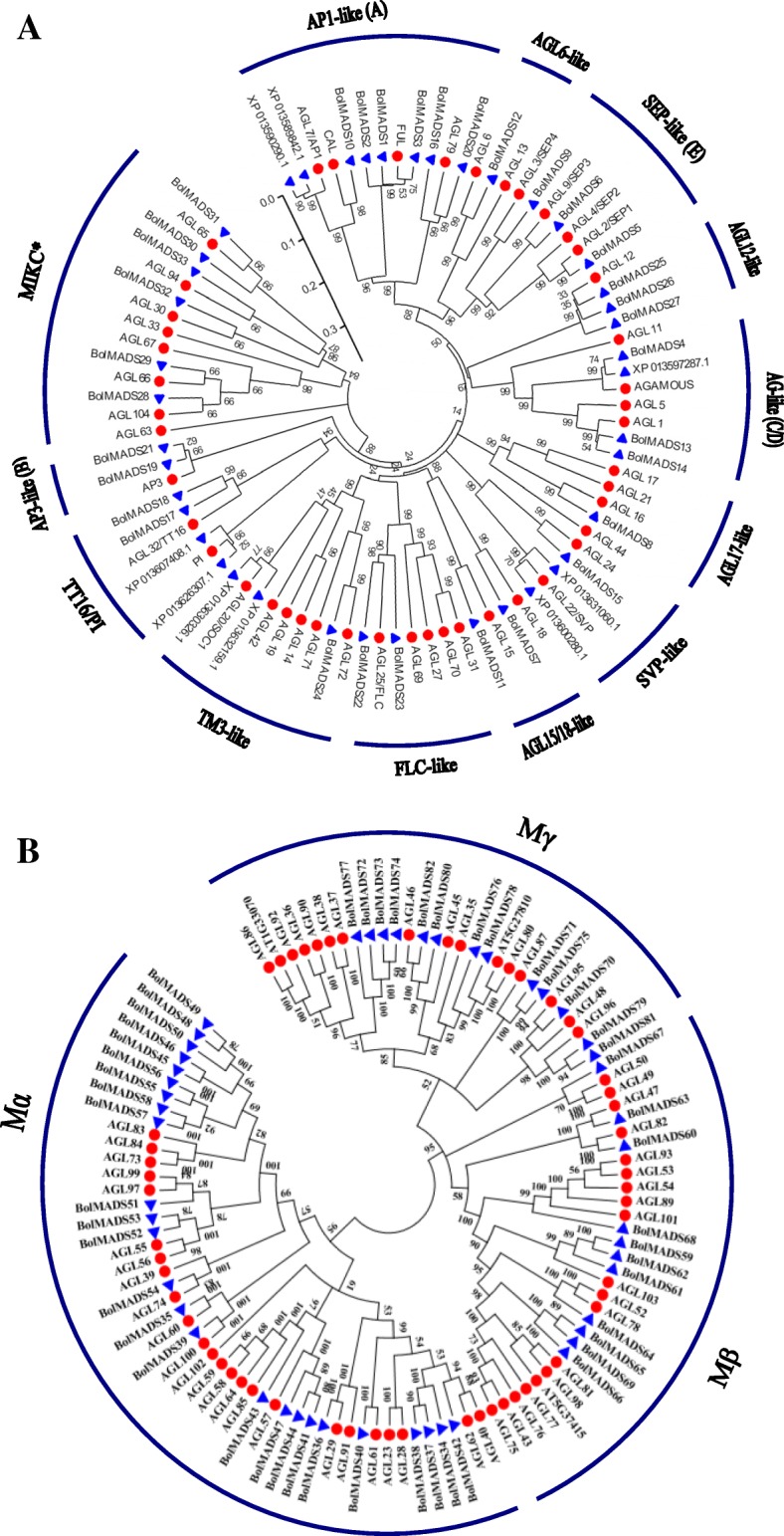
Phylogenetic tree of the sequence of full-length MADS-box proteins from *B. oleracea* and *A. thaliana* using neighbor-joining method. **a** Phylogenetic analysis of type II *B. oleracea* (42) and *A. thaliana* (45) MADS-box proteins classified into 12 MIKC^C^ clades and one MIKC* group as marked in the Fig. **b** Phylogenetic analysis of type I MADS-box proteins from *B. oleracea* (49) and *A. thaliana* (59)

A phylogenetic tree was constructed based on the sequence of full-length MADS-box proteins from *B. oleracea* and *A. thaliana* using the neighbor joining method (Additional file 1: Figure S1). The 91 BolMADS genes were classified into two categories, type I (49) and type II (42). Independent phylogenetic trees of both types were constructed using MADS genes from both species (Fig. 1). Type I *BolMADS* genes were further divided into three subgroups: Mα, Mβ, and Mγ. The Mα subfamily was the largest, possessing 25 genes, while the Mβ and Mγ subgroups showed nearly the same number of genes with 11 and 13, respectively.

In type II *BolMADS* genes, we found 36 MIKC^C^-type and 6 MIKC*-type genes. A total of 12 MIKC^C^ evolutionary branches were detected according to the known groups of *A. thaliana* MADS-box genes. The *AP1*-like branch contained the largest number (8) of *BolMADS* MIKC^C^-type genes. We found two subgroups of *AGL6*-like and *AGL17*-like genes which consisted of only one member each. In contrast with *A. thaliana*, *B. oleracea AP1*-, *AGL12*-, *TT16*/*PI*-, and *AP3*-like gene clusters are expanded by gene duplication, whereas *TM3*-, and *AGL17*-like genes do not have paralogs.

Using the neighbor joining method, another phylogenetic tree was constructed with the full-length of MADS-box protein sequences from *B. oleracea* and *B. rapa* (Additional file 2: Figure S2). In general, most MADS genes in *B. rapa* showed repetition and expansion compared to those from *B. oleracea*. Moreover, type II MADS-box genes showed higher expansion degrees than type I genes. In type II MADS genes, the TM3 subgroup showed the highest gene expansion degree, with a total of 16 *TM3* genes in *B. rapa* but only three in *B. oleracea*. The *AP3* and *AGL12* subgroups contained nearly the same number of genes in these two *Brassica species*.

### Gene structure and conserved motif distribution analysis

The gene structure and intron/exon arrangements of the *BolMADS* genes were determined by comparing their full-length cDNA and genomic DNA sequences. All type II *BolMADS* genes contained at least three introns except for *BolMADS16* and *BolMADS23*. *BolMADS16* showed the most conserved domain of M with no introns and *BolMADS23* showed two introns. In type I genes, *BolMADS34*, *42*, *67*, *78*, *80*, and *82* were found to have one intron, while the others were intronless (Table 1).

**Fig. 2:**
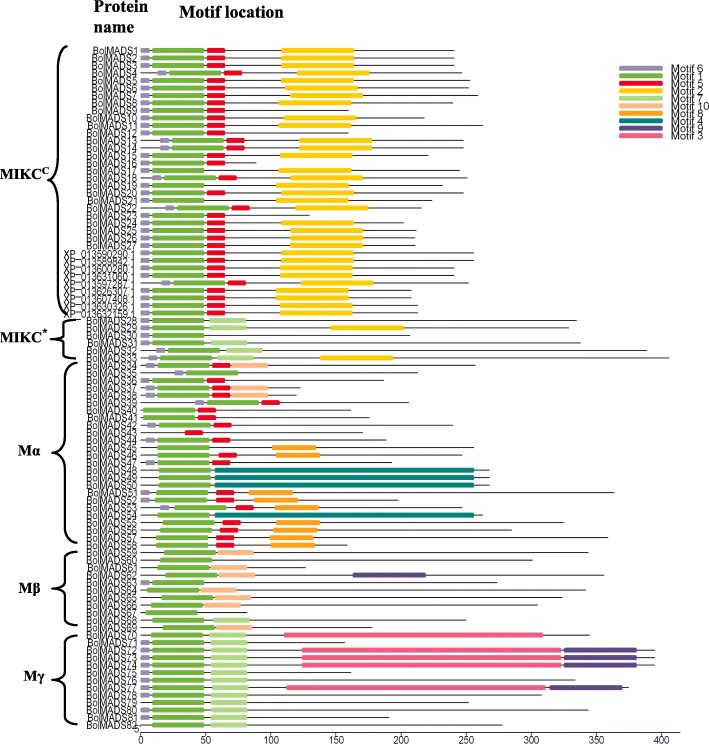
Distribution of conserved motifs in *B. oleracea* MADS-box proteins identified using MEME

The structures of proteins produced by *BolMADS* genes were analyzed using MEME online software. Ten conserved motifs, named as motifs 1–10, were identified (Fig. 2). Motif 1 and 5 were corresponding to the typical MADS domain. The main motif 1 was found in all BolMADS proteins. Motif 2 specified the K domain was found in most MIKC^C^ type genes except BolMADS9, 12, 16 and 23, which contained relatively short amino acid sequences. For the six MIKC^*^ type proteins, BolMADS29 and 33 contained motif 2, while the others have not. The K domains typical of type II proteins were missing in all type I BolMADS proteins. N-terminal motif 6 was found in all type II and in the majority of type I proteins. Motifs 4 and 8 were specific to some of the Mα proteins, motif 10 - to some of the Mα and Mβ proteins, while motifs 3 and 9 - to some of the Mγ proteins.

### Chromosome distributions of MADS-box genes

**Fig. 3:**
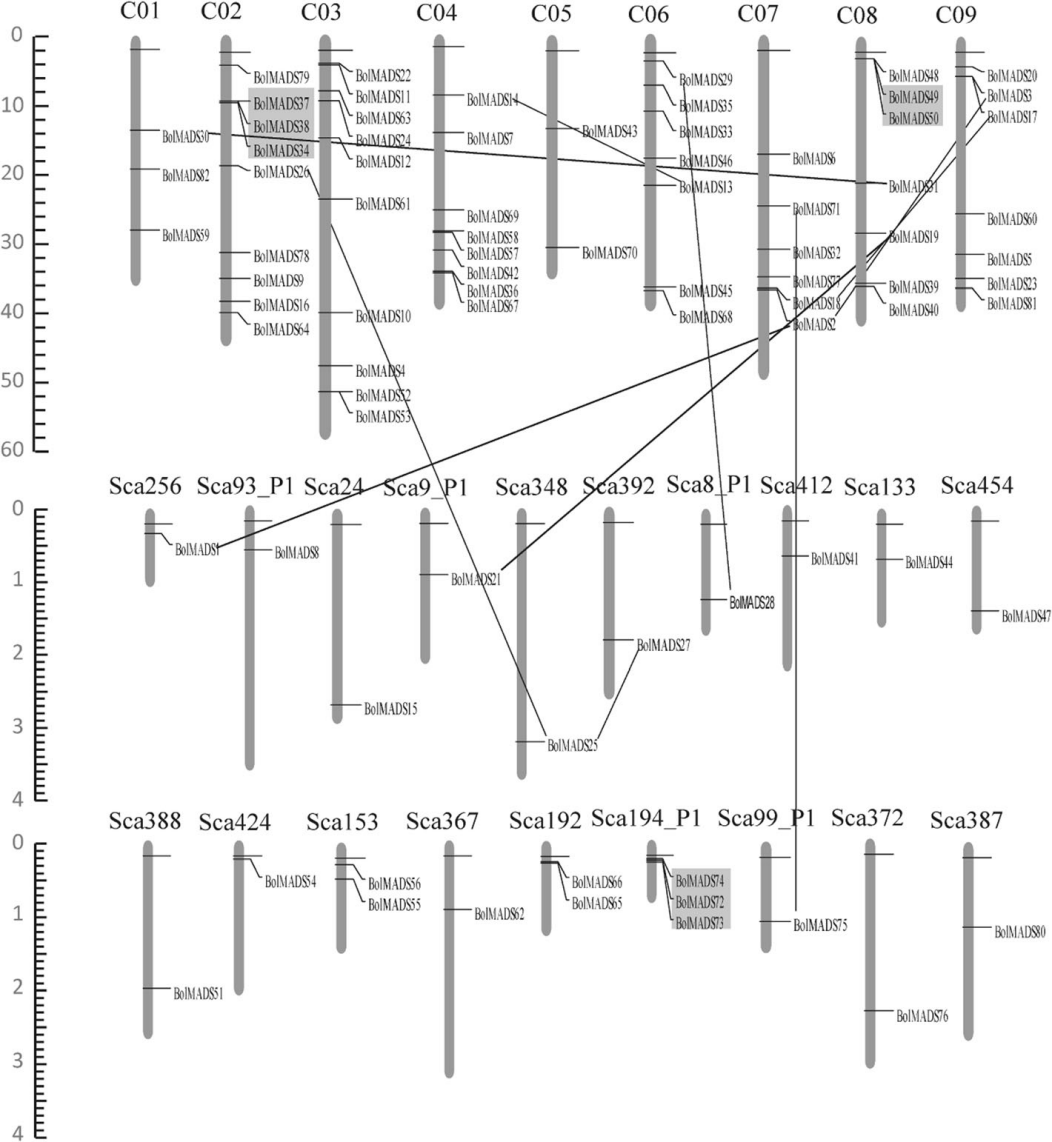
Distribution of *B. oleracea* MADS-box genes on 9 chromosomes and 19 scaffolds. Chromosome numbers are written as C01–C09 above each chromosome. The genes showing segmental duplications are indicated by black vertical lines. Tandemly duplicated genes are joined by a gray shadow. The size of each chromosome was estimated as Mb on the left side of the figures

The physical locations of *BolMADS* genes were mapped to the 9 chromosomes of *B. oleracea* (Fig. 3). Of the 91 genes, 59 were randomly distributed on 9 chromosomes, 23 were assigned to 19 scaffolds and 9 from the NCBI database had no location information. For MIKC-type genes, 26 were mapped on 8 chromosomes (except Chr5). Chromosomes 3 and 9 contained the maximum number of six genes, while only one gene was found on chromosome 1. The other 7 MIKC-type genes were randomly distributed on 7 scaffolds. Type I genes showed a completely different distribution. First, of the 49 type I genes, 31 scattered nonrandomly on all 9 *B. oleracea* chromosomes. Chromosomes 2 and 4 had the largest number of genes and the other chromosomes contained 2–5 genes. Second, the remaining 16 genes were distributed on 12 scaffolds. Sca194_P1 had the largest number of genes, showing three genes. Sca153 and Sca192 each contained two genes. There was one gene on each of the other scaffolds.

Duplicated genes including segmental and tandem duplications were detected in the MADS-box gene family of *B. oleracea*. There were higher frequencies of segmental duplications and a total of 10 genes contained corresponding homologous segments. In contrast, only three groups of genes were contained tandem duplications, which all related to type I genes. The first group containing *BolMADS37*, *38*, and *34* was on chromosome 2 and the second group containing *BolMADS49* and *50* was on chromosome 8. The third group contained three genes, *BolMADS 72*, *73*, and *74*, distributed on scaffold Sca194_P1.

### Genome-wide expression analysis of MADS-box genes

**Fig. 4:**
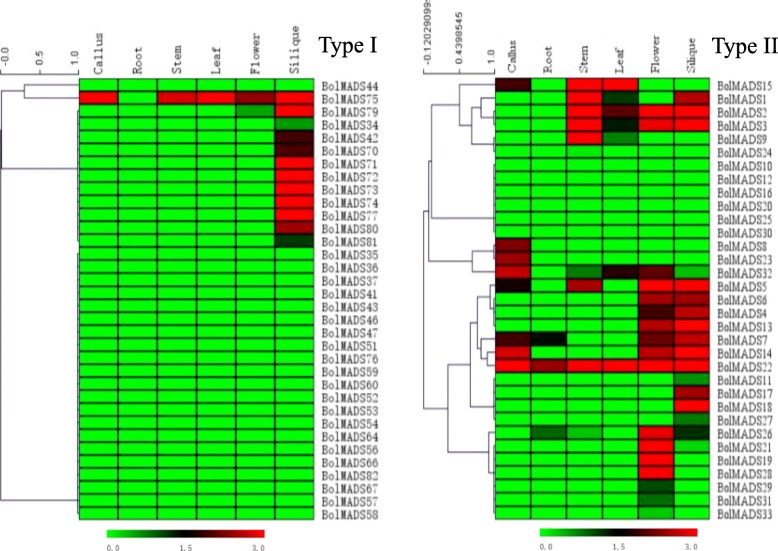
Heat map of *BolMADS* genes in the callus, root, stem, leaf, flower, and silique tissues. Hierarchical clustering results are shown on the left of the heat map and relative expression values with color green (low) to red (high) are displayed at the bottom

The RNA-seq data of 82 MADS-box genes were downloaded from *B. oleracea* Genomics Database (http://www.ocri-genomics.org/bolbase/) and the other 9 genes from the NCBI database had no information of RNA-seq. The genes were found to be differently expressed in six tissues including the callus, root, stem, leaf, flower, and silique. Two heat maps were constructed for type I and type II genes (Fig. 4). The overall expression of type II genes was more active and diverse than that of type I genes. A total of 30 genes showed no expression or their transcription levels were too low to be detected on the heat map. Of the 30 genes with no or low expression, 22 were type I and 8 were type II. In all six tissues, only the *FLC*-like gene *BolMADS22* was expressed.

Majority of MIKC-type genes (22 of 33), belonging to the *AP1*, *SEP*, *AG*, *AGL12*, *AGL15/18*, and *FLC* subfamilies, were expressed in the flower and /or silique. *BolMADS19* and *21* (*AP3*-like) were consistently and specifically expressed in flowers. However, most genes within one subfamily often showed different expression patterns. Among the six *AP1* genes, three (*BolMADS1*, *2*, *3*) showed high expression levels in the flower and silique and three genes (*BolMADS10*, *16*, *20*) showed no expression in the six tissues. In the *AGL12*-like subfamily containing three genes, *BolMADS26* was highly expressed in the flower, while the other two genes showed no or very low transcription in the six tissues. Interestingly, six MIKC^*^ genes also displayed very different expression patterns. Four genes (*BolMADS29*, *30*, *31*, *33*) showed no or low expression levels. The other two genes (*BolMADS28*, *32*) were highly expressed in flowers and *BolMADS32* was detected in the leaf and callus. Compared to MIKC genes, type I genes were inert and their expression patterns were simple. A transcription of only 12 genes was detected, while the other genes were silent. These 12 genes were expressed in the silique and *BolMADS75* showed high expression in the flower, leaf, stem, and callus.

### Expression patterns of MADS-box genes in different tissues of cauliflower

**Fig. 5:**
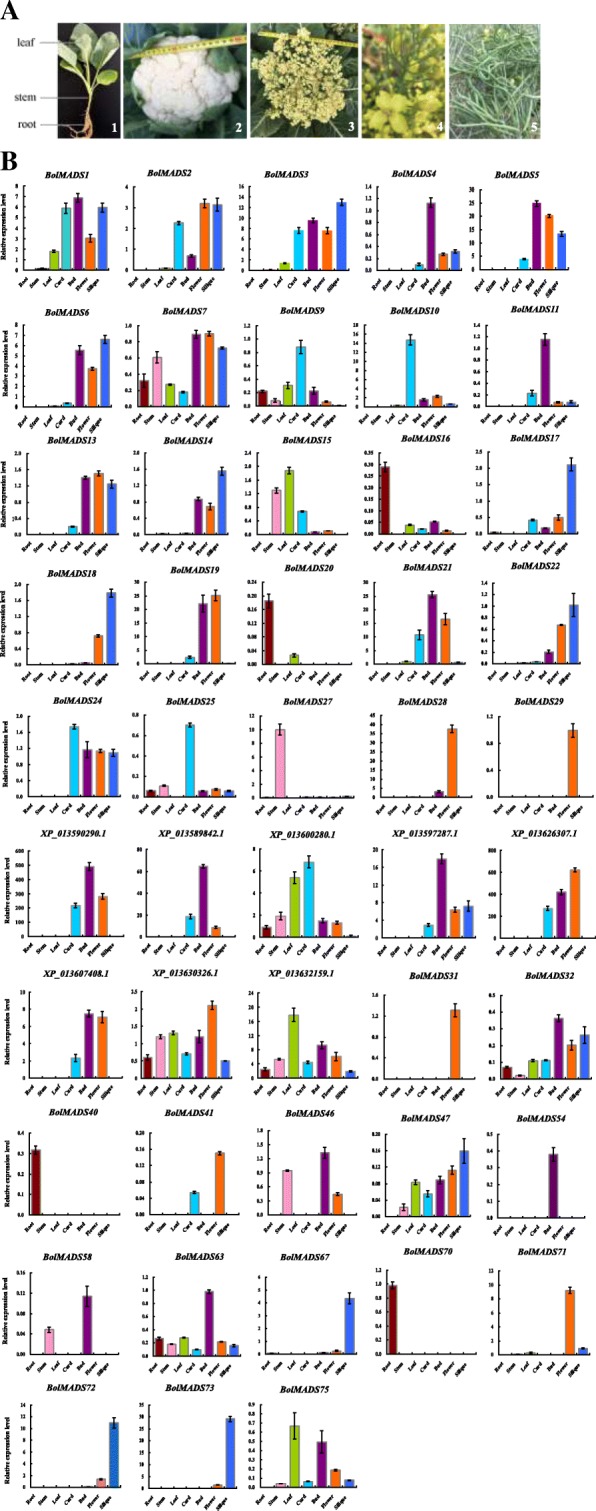
Expression patterns of 48 *BolMADS* genes in cauliflower tissues of root, stem, leaf, curd, bud, flower, and silique (**a**) by qRT-PCR (**b**). A1, The tissues of root, stem and leaf from 35-day-old plant; A2, The curd of 60 days after transplanting; A3, The bud of 90 days after transplanting; A4, The fully opened flowers during the flowering period; A5, The siliques of 20 days after pollination. The standard deviations of three biological replicates are represented by the error bars

MADS-box genes were reported to have diverse functions in plant growth and development, particularly in floral organs specification. Therefore, we examined the expression patterns of 87 *B. oleracea* MADS-box genes in seven different tissues of cauliflower including the root, stem, leaf, curd, bud, flower, and silique by qRT-PCR (Fig. 5). A total of 48 genes were expressed in at least one tissue, while the other 39 genes showed no or very low expression. Among the 48 expressed genes, 35 belonged to the MIKC type and the other 13 were type I.

All MIKC type genes were highly expressed in reproductive organs including the curd, bud, flower, and silique except for *BolMADS15* (*SVP*-like), *16* (*AP1*-like), *20* (*AP1*-like), and *27* (*AGL12*-like), which showed higher transcription levels in vegetative tissues (root, stem, and leaf). We found that not only *AP1*-like genes (*BolMADS1*, *3*, *10, XP_013590290.1, XP_013589842.1*), but also *AP3*-like *BolMADS21* gene, *SEP1*-like *BolMADS5* gene, *SVP*-like *XP_013600280.1* gene and *PI*-like *XP_013626307.1* gene showed relatively high expression levels in the curd, which is a specific reproductive organ in cauliflower. This result indicates that these genes play important roles in cauliflower curd formation and also confirmed the results of a previous study showing that *AP1*-like genes control flower primordium development. In addition, *XP_013590290.1* and *XP_013589842.1* were also highly expressed in the bud and flower, and *XP_013590290.1* showed the highest expression level in the flower. The six MIKC^*^ genes showed no or relatively low expression except *BolMADS28*, which was expressed in flower with relatively high transcription level among MIKC type genes.

For the 45 tested type I genes, 13 showed different expression patterns. *BolMADS71* showed the highest expression level in the flower. *BolMADS40* and *70* were specifically expressed in the root, indicating roles in root development. The other three genes (*BolMADS67*, *72*, and *73*) showed high transcription levels in the silique and may be involved in silique development.

### Key MADS-box genes involved in floral organ development

**Fig. 6:**
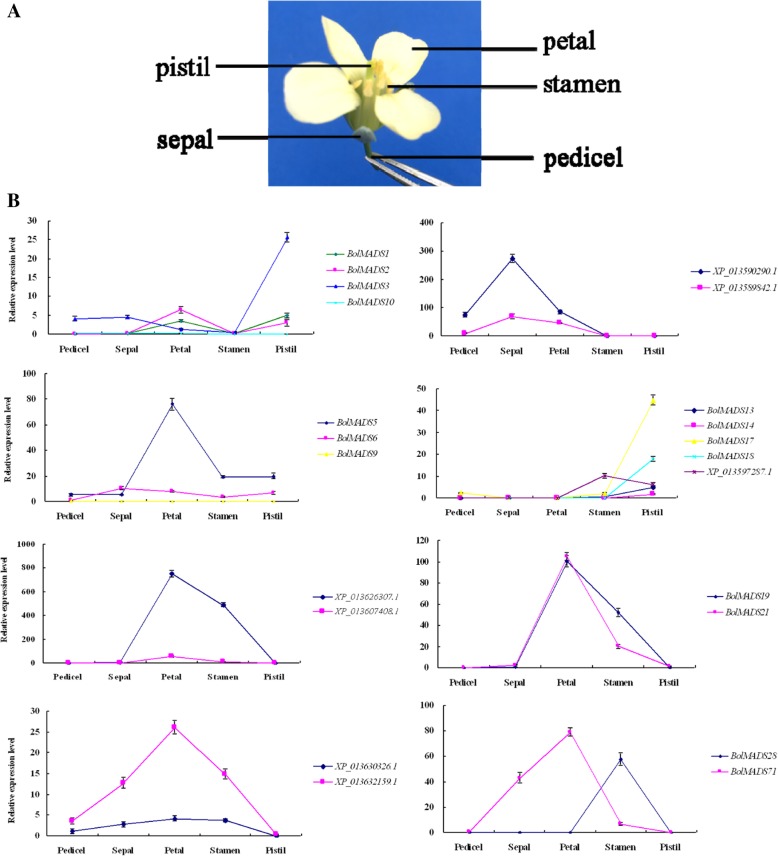
Expression profiles of 22 selected *B. oleracea* MADS genes in pedicel and four whorls of the flower including the sepal, petal, stamen, and pistil (**a**) based on qRT-PCR (**b**) analysis. The standard deviations of three biological replicates are represented by the error bars

A total of 21 MIKC type genes and one type I gene showing relatively high expression levels in the flower were selected to further investigate their roles in floral organ development. The expression patterns of these genes were detected in *B. oleracea* pedicel, sepal, petal, stamen, and pistil by qRT-PCR (Fig. 6). Three *AP1*/*FUL*-like (*BolMADS1*, *2*, *3*), two *AP1*/*AP1*-like (*XP_013590290.1* and *XP_013589842.1*) and one *AP1*/*CAL*-like (*BolMADS 10*) orthologous genes showed different expression modes. *BolMADS1*, *2*, and *3* showed relatively higher expression levels in the pistil than in other flower organs, while *BolMADS10* showed low expression in all five flower organs. *XP_013590290.1* and *XP_013589842.1* highly expressed in sepal and petal, and *XP_013590290.1* showed the highest expression level in sepal. *AP3*-like *BolMADS19* and *21* and *PI*-like *XP_013626307.1* and *XP_013607408.1* were characterized as B class genes. These two groups of genes were specifically expressed in the petal and stamen and *XP_013626307.1* showed the highest expression level in these two floral organs. *BolMADS13* and *14* of the *AG*-like subfamily (C/D class) showed a similar expression pattern as *BolMADS17* and *18* from the *GGM13*-like subfamily, which were characterized as B sister genes (Bs class). These two groups of genes were preferentially expressed in the pistil. In addition to expression in the pistil, another *AG*-like *XP_013597287.1* gene also demonstrated trace expression in the stamen. *BolMADS5* and *6* belonging to the *SEP*-like subgroup (E class) were detected in all four floral organs. The above results are consistent with the classical ABCDE flower model.

*XP_013630326.1* and *XP_013632159.1* were *SOC1* homologous gene belonging to the *TM3*-like subfamily. These two genes were expressed in all the five flower organs except the pistil. The overall expression level of *XP_013632159.1* was much higher than that of *XP_013630326.1*. *BolMADS28* of the MIKC^*^ type was specifically expressed in the stamens and its expression level was the second highest among all *B. oleracea* MADS-box genes, indicating its involvement in stamen development. One type I gene, *BolMADS71*, was preferentially expressed in the petal at relatively high levels. This indicates that *BolMADS71* also participates in the process of flower development.

## Discussion

Cauliflower is one of the most important vegetables grown worldwide. Curd formation, floral organ development, and flowering time are important agronomic traits in cauliflower breeding and production for directly determining its adaptability and commerciality [36]. The MADS-box genes, particularly the MIKC family members, play important roles in plant flower development. Previous studies reported the characterization of MADS-box genes in several Cruciferous crops including *A. thaliana* and *B. rapa* [15, 43]. With the completion of the *B. oleracea* genome sequence, the *BolMADS* genes can be systematically identified and analyzed [42]. In this study, we identified a total of 91 *BolMADS* genes and comprehensively analyzed these genes to determine their phylogenetic relationships, chromosomal locations, gene structures, and expression patterns.

### Causes of MADS-box genes loss in *B. oleracea*

Approximately 20–40 Mya, one whole genome duplication (WGD) event occurred in the Brassicaceae genome, after which the *B. oleracea* genome underwent another two whole genome triplication (WGT) events against *Arabidopsis* [44–46]. This caused the *B. oleracea* genome to become nearly 5-fold larger than the *Arabidopsis* genome. However, the number of MADS-box genes in *B. oleracea* is not directly proportional to the genome size. Only 91 *BolMADS* genes were identified, which is lower than the numbers in *A. thaliana* and *B. rapa* [15, 47]. This may be for two reasons. First, after genomic duplication, plants often show large areas of gene loss and chromosome rearrangements to maintain a metabolic balance [45]. Thus, most *BolMADS* genes may have been lost through later evolutionary processes. Second, approximately 4 million years ago, *B. oleracea* was differentiated as a branch [46]. Since then, this species has been subjected to strong artificial and natural selection, resulting in the loss of different *BolMADS* genes.

### Organization of BolMADS genes

Gene organization was reported to play important roles in the evolution of multigene family [48–50]. In this study, the MIKC type genes contained a much larger number of introns compared to M-type genes. Among the MIKC type genes, 30 of 33 contained more than 3 introns, whereas all M-type genes had no or only one intron. The same intron distributions have been observed in other species, such as *Arabidopsis*, Chinese cabbage, rice, and cotton [15, 45, 51, 52]. This can be interpreted as a difference between type I and II MADS genes in their tendencies for deletion or acquisition of introns and indicate evolutionary conservation between different plant species [53]. *BolMADS* genes within the same group had a similar motif construction with common evolutionary ancestors. The consistency in intron number, motif structure, and phylogeny demonstrate that these genes were correctly classified.

### Expression patterns of MIKC type genes in cauliflower

Most MIKC type genes were not expressed or lowly expressed in vegetative organs, including the roots, stems, and leaves, and were abundantly expressed in the reproductive organs, suggesting that MIKC type genes play important roles in the growth and development of reproductive tissues.

The classical model of flower development in plant is the ABCDE model. In *A. thaliana*, four A class genes have been cloned: *AP1*, *CAL*, *FUL*, and *AGL79*. *AP1* acts as a floral organ identity gene to promote the development of petals and sepals and specifies floral meristem identity [16, 54]. However, *CAL*, which is an *AP1* paralogue, has only partial functions of *AP1* and promotes floral meristem formation during flower development [19, 20]. In cauliflower, two *AP1* (*XP_013590290.1* and *XP_013589842.1*) and one *CAL* homologous genes (*BolMADS10*) were detected and they were highly expressed in the curd tissue. Curd is the edible organ of cauliflower, which is composed of inflorescence meristems. This result indicates that these three genes play important roles in curd formation. *FUL* has a wide range of functions: it can play a role in the flower organ specification and function in carpel development [55, 56]. A total of three *FUL* homologous genes (*BolMADS1*, *2*, and *3*) were detected and showed similar expression patterns with relatively higher expression in the petals and ovary than in the other floral organs.

The main function of the B class genes (*AP3* and *PI*) is to control the development of stamens and petals, particularly in the decision to form male reproductive organs [21]. Two *AP3*-like (*BolMADS19* and *21*) and two *PI*-like (*XP_013626307.1* and *XP_013607408.1*) B class genes were detected in cauliflower and were highly expressed in the petals and stamens, indicating similar functions.

A total of 4 genes in *Arabidopsis* belong to the C/D class, including *AG*, *SHP1* (also known as *AGL1*), *SHP2* (also known as *AGL5*), and *STK* (also known as *AGL11*). These genes are mainly involved in the development of stamens, carpels, ovules, and fruit [4, 22, 23]. The *BolMADS4*, *13*, *14* and *XP_013597287.1* genes of the C/D class were preferentially expressed in the pistil, indicating their participation in pistil development.

E class genes (*SEP*) have obvious partially redundant functions during flower development. Moreover, these genes do not function alone in flower development, but function through interactions with ABC genes. In different species, *SEP*-like genes have different degrees of sub-functionalization and neo-functionalization [25, 57]. In cauliflower, three *SEP*-like genes were identified, with *BolMADS5* showing the highest overall expression level in the flower. *BolMADS5* was expressed in all five floral organs, but its expression level was highest in the petals, followed by in the ovary and stamen, indicating its roles in the development of these three floral organs.

Interestingly, *BolMADS28* (MIKC^*^ type) was specifically expressed in the stamens and its expression level was highest among all MIKC type genes. Its homologue in *Arabidopsis* is *AGL104*, which is required for pollen maturation and pollen tube growth [58]. *BolMADS28* may be a new gene that plays an important role in stamen development in cauliflower.

### Expression profiles of M type genes in cauliflower

Compared to MIKC MADS-box genes, the information about functions of M type genes are very limited. Several studies in *Arabidopsis* indicated that some M type genes participate in plant growth and reproduction, particularly in defining the female gametophyte and in the development of the embryo and endosperm [58–60]. In this study, 13 of 45 (28.89%) M type genes were detected in different tissues. Most detected M type genes were mainly expressed in reproductive tissues, while other several genes showed specific expression in the roots, such as *BolMADS40* and *70*. *BolMADS71* showed the highest expression level in the flower tissue, mainly in the petals. This suggests the participation of *BolMADS71* in petal development. *BolMADS67*, *72*, and *73* were the top most abundantly expressed genes in the silique, indicating their roles in silique development.

## Conclusions

In conclusion, 91 genes were identified in the *Brassica oleracea* genome. These genes were divided into 42 type II and 49 type I genes, and the type II genes were further divided into 13 subfamilies. The exon/intron structures, conserved motif distributions, and chromosomal locations of MADS-box family members in *B. oleracea* were also determined. Expression analysis of the *BolMADS* genes indicated that most MIKC^C^ genes participated in flower development, which is consistent with the ABCDE model. Several non- MIKC^C^ genes were also found to be highly expressed in the stamens and petals, indicating their important roles in the development of these floral organs.

## Methods

### Identification of MADS-box gene family in *B. oleracea*

The sequences of Arabidopsis MADS-box genes were obtained from the TAIR database (http://www.arabidopsis.org) and used as queries for a BLASTP algorithm-based against the *B. oleracea* genome database (http://www.ocri-genomics.org/bolbase/). For all candidate genes, we also examined whether they contain the MADS domain (PF00319) in the SMART (http://smart.embl-heidelberg.de) and Pfam (http://pfam.sanger.ac.uk) databases. Sequences without a MADS domain were deleted.

### Conserved motifs and gene structure analysis of MADS-box proteins

Conserved motifs in MADS proteins of *B. oleracea* were identified using the program Multiple Em for Motif Elicitation (http://meme-suite.org/tools/meme). Default parameters were selected except that the maximum number of motifs was set to 10. The *BolMADS* gene structure was predicted using the program of GSDS2.0 (Gene Structure Display Server, http://gsds.cbi.pku.edu.cn/) for both genome and coding domain sequences.

### Phylogenetic tree construction and protein conserved domain sequence alignment

MADS proteins of *B. oleracea* with *A. thaliana* and *B. rapa* [47] were aligned respectively using ClustalW with default settings. The two phylogenetic trees were constructed with MEGA5.04 software using the neighbor-joining method. The bootstrap test was executed by 1000 replications. The resulting phylogenetic tree was prepared in FigTree (v1.3.1) software.

### Chromosomal location and gene duplication

The physical positions of all MADS-box genes along each chromosome were identified from the *B. oleracea* database. Gene duplication information was obtained by aligning all *B. oleracea* MADS-box genes. The criteria of both the query coverage rate and identity of the aligned region were more than 80%.

### Plant materials and tissue-specific expression analysis

In this study, RNA-seq data from the *B. oleracea* Genomics Database (http://www.ocri-genomics.org/bolbase/) were used to analyze the expression patterns of MADS-box genes in different tissues of cultivated *B. oleracea* plants.

Seven types of tissues from cauliflower (a DH line from cauliflower cultivar “QingNong 65”) were used for quantitative real-time (qRT)-PCR analysis of all MADS-box genes. These tissues included the root, stem, leaf, curd, bud, flower, and silique. MADS-box genes with relatively higher transcription levels in the flower tissue were selected for qRT-PCR analysis in pedicel and four whorls of flower organs, including the sepal, petal, stamen, and pistil.

Approximately 100 mg of frozen tissue was collected for total RNA isolation using an RNA kit (RNAprep Pure Plant Kit, Tiangen, China) according to the manufacturer’s instructions. One microgram of total RNA was reverse-transcribed to the first-strand cDNA using the PrimeScript RT reagent Kit. Specific primers for all MADS-box genes were designed using Primer-Blast online software from NCBI (http://www.ncbi.nlm.nih.gov/tools/primer-blast/, Additional file 3: Table S1).

qRT-PCR assays were conducted in an ABI StepOnePlus machine using SYBR® Premix Ex Taq™ (TaKaRa, Shiga, Japan). The reaction mixture included 12.5 μl of 2 × SYBR Green PCR Master Mix (Applied Biosystems), a diluted cDNA template of 30 ng, 500 nmol of each primer in a final volume of 25 μl. The PCR conditions were as follows: 95 °C for 10 min, 40 cycles of 10 s at 95 °C, 50 °C for 15 s, and 72 °C for 30 s. The amplification efficiency of each primer pair was performed according to the method described by our previous study [61]. The 2^−ΔCt^ value was used to measure the relative expression levels of specific genes. ACT and EF1a were used as reference genes. Each sample was analyzed in three biological and three technical replicates.

## Additional files

Additional file 1: **Figure S1.** Phylogenetic tree of MADS-box genes from *B. oleracea* and *A. thaliana* using neighbor-joining method. (DOC 414 kb)

Additional file 2: **Figure S2.** Phylogenetic tree of MADS-box genes from *B. oleracea* and *B. rapa* using neighbor-joining method. (DOC 1007 kb)

Additional file 3: **Table S1.** The specific primers for qRT-PCR of 87 MAD-box genes. (DOC 215 kb)

## Abbreviations

*AGL*: *AGAMOUS*-LIKE
*AP1*: *APETALA1*
*PI*: *PISTILLATA*
*SEP*: *SEPALLATA*
WGD: whole genome duplication
WGT: whole genome triplication
