# Supplementary material for: Genome wide analysis of MADS-box gene family in Brassica oleracea reveals conservation and variation in flower development
Source: BMC Plant Biol. 2019 Mar 19;19:106. doi: 10.1186/s12870-019-1717-y (PMC6425688; doi:10.1186/s12870-019-1717-y)
Supplement: Supplementary file 2 — Figure S2. Phylogenetic tree of MADS-box genes from B. oleracea and B. rapa using neighbor-joining method. (DOC 1007 kb) [file 12870_2019_1717_MOESM2_ESM.doc]

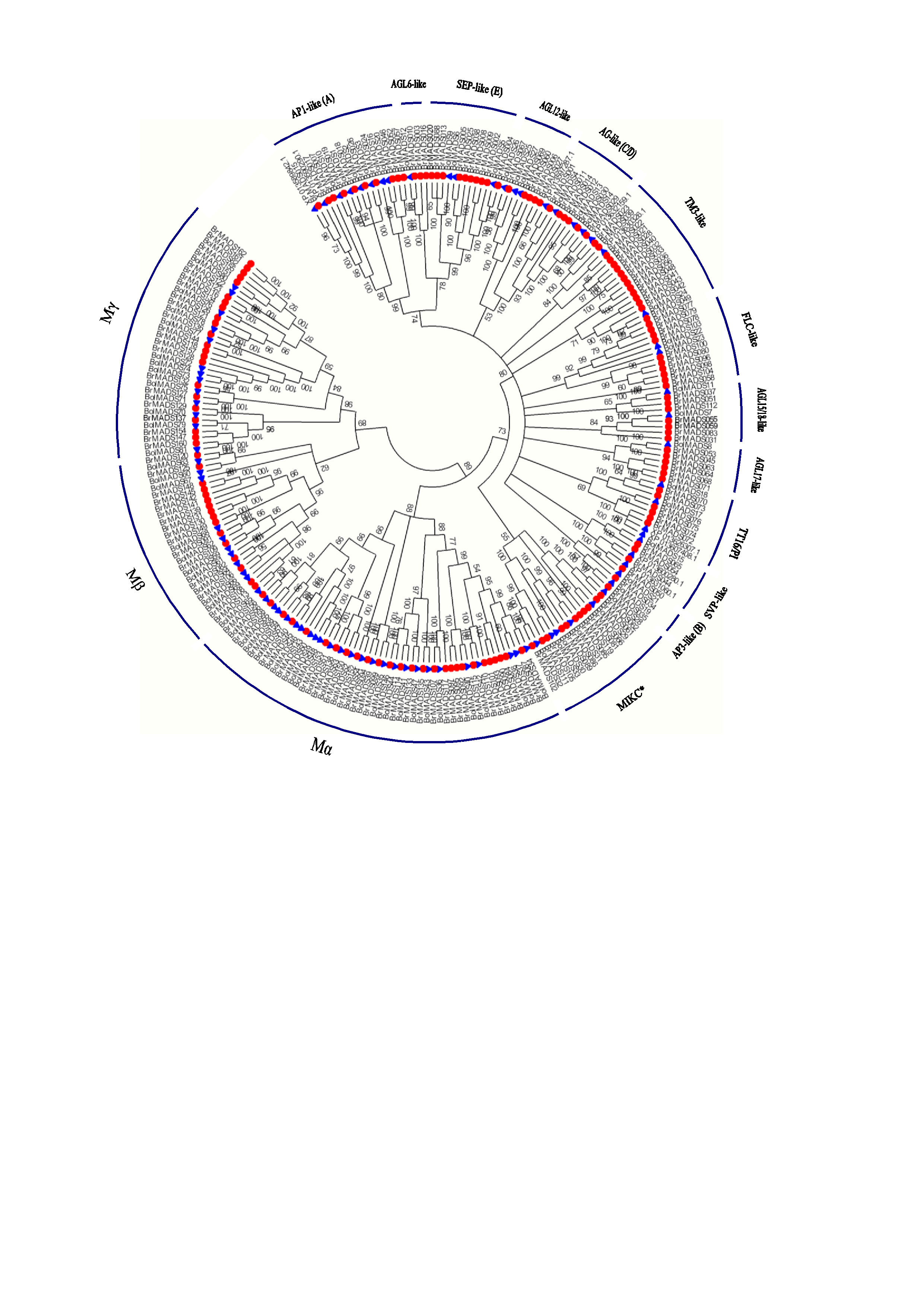


**Figure S2** Phylogenetic tree of MADS-box genes from *B. oleracea* and *B. rapa* using neighbor-joining method.
