# Supplementary material for: Genome wide analysis of MADS-box gene family in Brassica oleracea reveals conservation and variation in flower development
Source: BMC Plant Biol. 2019 Mar 19;19:106. doi: 10.1186/s12870-019-1717-y (PMC6425688; doi:10.1186/s12870-019-1717-y)
Supplement: Supplementary file 3 — Table S1. The specific primers for qRT-PCR of 87 MAD-box genes. (DOC 215 kb) [file 12870_2019_1717_MOESM3_ESM.doc]

Table S1 The specific primers for qRT-PCR of 87 MAD-box genes

| **Gnen name** | **Gene locus** | **Primer sequences (5’-3’)** | **PCR efficiency (%)** |
| --- | --- | --- | --- |
|  |  |  |  |
| BolMADS1 | Bol007763 | CCAAGCTCAAGGCAAGAGTT | 98.3 |
|  |  | TGGATTCGAACATAGCTTGG |  |
| BolMADS2 | Bol017059 | AGATCACAACAATGCGCTTC | 99.5 |
|  |  | GCCATCTCTGGAGGAGGTTA |  |
| BolMADS3 | Bol036265 | AGCGCTCCAGAAGAAGGATA | 97.4 |
|  |  | ATCTCTGGTGGAGGTTACGC |  |
| BolMADS4 | Bol029887 | AACACTGGATCGGTAGCACA | 101.2 |
|  |  | TTTGGATCGTATTCGGTTGA |  |
| BolMADS5 | Bol030482 | GGGAAGGCAATGAACAGAAT | 99.6 |
|  |  | GTTGCAGTTATCTGCTCCGA |  |
| BolMADS6 | Bol041762 | GATCCGGACACTGGAGAGAT | 103.4 |
|  |  | TAAGGCTTCGTAACGCTCCT |  |
| BolMADS7 | Bol011058 | TGAGGAGCGAACTTGAGAGA | 96.8 |
|  |  | TTTCGATCCTTGACGCTATG |  |
| BolMADS8 | Bol024219 | ATTCTCTGCGATGCTGAGGT | 99.3 |
|  |  | TCATTTCCGAATTGGTGTCA |  |
| BolMADS9 | Bol020052 | TGACCAAGACGGTTGAGAAG | 101.2 |
|  |  | CTCAAGCTCATTCACTCCCA |  |
| BolMADS10 | Bol024957 | CACGAGTCCCTCAACCACCT | 100.6 |
|  |  | TGTAAGGATTTAACTGCGGTTGCG |  |
| BolMADS11 | Bol034251 | TCCTACATCAAATGCTTCGC | 98.7 |
|  |  | GATGGGCTCTCTCTGTCTCC |  |
| BolMADS12 | Bol029555 | CATGTAATTGGTGCCAGGAG | 99.3 |
|  |  | CAGTAAGAGCGGCTTCCAG |  |
| BolMADS13 | Bol023763 | GCAGCAGGATTCGAGTGTAA | 96.9 |
|  |  | GGTCTTGACCGGAGAAATGT |  |
| BolMADS14 | Bol004959 | GATCCGGAACAACAGGAATC | 99.5 |
|  |  | CCGGAATATAGCTCCGGTTA |  |
| BolMADS15 | Bol037657 | GCTGGAGAAATCGCTTGAAT | 102.6 |
|  |  | CTCCTTTAACGCCATCGTTT |  |
| BolMADS16 | Bol033138 | TGATGCTGATGTTGCTTTGA | 101.3 |
|  |  | TTGAAGAAATTCAAACCTGGAG |  |
| BolMADS17 | Bol036252 | TCATCATCAGCAACAAAGCA | 100.4 |
|  |  | AACCTTGGTGATCGTTGGAT |  |
| BolMADS18 | Bol017090 | AAGAGGCGGATGCTAGAAGA | 101.2 |
|  |  | GTTTGGTTGTTGTTGCTGCT |  |
| BolMADS19 | Bol025193 | TGGAGCTAAGGGCAGAAGAT | 97.3 |
|  |  | AATGGTGATGCTGGTTCTGA |  |
| BolMADS20 | Bol032290 | CAAACCCTCAACCAAGGATT | 100.4 |
|  |  | TTCGTTCCTTTGCAATGTGT |  |
| BolMADS21 | Bol041644 | AATCGCTTGGGAATCAAATC | 95.8 |
|  |  | TTGGGATAATGGTGGTGATG |  |
| BolMADS22 | Bol008758 | CAAATGTCGGTGGTGTAAGC | 96.8 |
|  |  | GCCAAAGCCTGATTCTCTTC |  |
| BolMADS23 | Bol043693 | CGATATGGAAAGCAACATGG | 99.2 |
|  |  | CGGATCGAAACTTAAACCGT |  |
| BolMADS24 | Bol028071 | CATCACCAGAAGTTGATGGG | 100.9 |
|  |  | CCGCTTGGTTCTTCTCCTAC |  |
| BolMADS25 | Bol003519 | ATGTTTGGAGGAGGAGATGG | 96.2 |
|  |  | CGAGGAGATACTTGTTGGCA |  |
| BolMADS26 | Bol034952 | TCGAGATGCTTCAGAAAGGA | 99.4 |
|  |  | TTTCACTTGGCATGGTTAGC |  |
| BolMADS27 | Bol002366 | TGGTCGTGGTTCTTCTTCTG | 97.6 |
|  |  | GCAGAGCGAATATGGGAAAT |  |
| BolMADS28 | Bol042006 | ATCGGCCCATTCTAATCAAC | 97.3 |
|  |  | TGGCATTCGCTCATATTGTT |  |
| XP_013590290.1 | XP_013590290.1 | CTGCCCTTAAGCACATCCGA | 98.6 |
|  |  | CATGGTTCTGCTGGTCCCAT |  |
| XP_013589842.1 | XP_013589842.1 | GACTTGCAAGCAATGAGCCC | 99.5 |
|  |  | CATGGTTCTGCTCGTCCCAT |  |
| XP_013600280.1 | XP_013600280.1 | AGAGATTGCGGGAAAGAGCC | 99.3 |
|  |  | TGTGTCCCTTGCTGCCTTAG |  |
| XP_013631060.1 | XP_013631060.1 | ACGCGCGTGATTGAAACAAA | 98.3 |
|  |  | CCTAGTCGCTCGTTCTCGTC |  |
| XP_013597287.1 | XP_013597287.1 | CCACAGAGGAAAGCTGGGAG | 97.6 |
|  |  | AGTGCGACTTCAGCATCACA |  |
| XP_013626307.1 | XP_013626307.1 | CCTCGACAAAGTCCGAGACC | 98.4 |
|  |  | ATTTGGCTGAATCGGCTGGA |  |
| XP_013607408.1 | XP_013607408.1 | TAGAGCACGCCGTTGAACAT | 99.2 |
|  |  | TTGGCTGAATCGGCTGAACT |  |
| XP_013630326.1 | XP_013630326.1 | AGCAGCTCAAGCAGAAGGAG | 97.6 |
|  |  | GCCACCTCGTCCACTTTCTT |  |
| XP_013632159.1 | XP_013632159.1 | TCGCCAGCTCCAATATGCAA | 98.1 |
|  |  | CCTGTGCCTTCTCCCAAGAG |  |
| BolMADS29 | Bol027523 | TACTACGGGCCTCAATCCTT | 99.5 |
|  |  | GAGACTTCGTGGTCTGCTTG |  |
| BolMADS30 | Bol013125 | GAAACAGCCAACTTATGCCA | 98.3 |
|  |  | CCTCCCATCTCCAAAGGTAA |  |
| BolMADS31 | Bol013192 | TCACTCGATGTCTTGGCTTC | 99.7 |
|  |  | TTGATCTTCGTCCATTCCAA |  |
| BolMADS32 | Bol007216 | GAATCATCACCAAACGTTGC | 97.3 |
|  |  | CATGGAGATAGAGCAAGGCA |  |
| BolMADS33 | Bol023922 | AACTGGCCTTCGTCTGAGAT | 102.4 |
|  |  | CTGGAGATAAGCCAGCAACA |  |
| BolMADS34 | Bol008953 | TCGAGGTCTGAACAATCGTC | 98.7 |
|  |  | AAAGCCAGGATGAGGAAATG |  |
| BolMADS35 | Bol026349 | GCTTTGAATGGGTTAAGGGA | 99.1 |
|  |  | TCATCGCTTACGGATTTGAG |  |
| BolMADS36 | Bol037864 | GATGATGATTCCGACAGTGG | 96.9 |
|  |  | GCTTCTCGAACCTCTCATCC |  |
| BolMADS37 | Bol008951 | TGCTCGTTTGGTCATACAAAG | 98.2 |
|  |  | TGGATTTCACCAAGTTGCAT |  |
| BolMADS39 | Bol009782 | TTCCGGTGAACTTGGATGTA | 99.3 |
|  |  | TATCCATCTGTGGCTTCCAA |  |
| BolMADS40 | Bol031566 | TTTCAGCAGCAAAGTCAACC | 97.4 |
|  |  | ACCGACCATTTAGCCATCTC |  |
| BolMADS41 | Bol001956 | TGATTTCGCTGAAGAGTTGG | 95.8 |
|  |  | CAGATGAACCATCACCGTCT |  |
| BolMADS42 | Bol033229 | AATCTGAGGATGGATTTGGG | 95.2 |
|  |  | GGATCAAACACCTCAGGCTT |  |
| BolMADS43 | Bol022502 | TGAAGATGGACGAGTTCTGC | 101.4 |
|  |  | TCTCCTCTTCGTCGTCCTTT |  |
| BolMADS44 | Bol019111 | ATGAACCGTCTGGGAAAGAC | 100.6 |
|  |  | TTCCCAACCTCTGTAAAGCC |  |
| BolMADS45 | Bol044870 | TTGTCAACTCGAAGAATCCG | 98.4 |
|  |  | AGGTCCATATCGCTCCATTC |  |
| BolMADS46 | Bol015619 | CACCTGACTTGGATGAATGG | 100.3 |
|  |  | CCCAAGCTTCCTGCTTCTAC |  |
| BolMADS47 | Bol001334 | AACCAGGACCTCATGGAAAG | 96.8 |
|  |  | TCTTCTCTTCCACGCTCAAA |  |
| BolMADS48 | Bol023453 | ACCAAACCCTAATCGCTACG | 95.4 |
|  |  | GCAGGTGATAACAGTGCGTT |  |
| BolMADS51 | Bol002451 | TTTACTTGGAAGCGTGGATG | 96.9 |
|  |  | TCAGAAACCGCTACCATCTG |  |
| BolMADS52 | Bol014335 | AGGCTTTGAAAGATCGGAGA | 97.1 |
|  |  | TCGTTGTTGTTTGTTTCGGT |  |
| BolMADS53 | Bol014328 | CGGGCTCCTAAGATTCGTAA | 99.8 |
|  |  | CAGAACCAGAAGCACCTTCA |  |
| BolMADS54 | Bol001745 | CTCTCGACGGATCTTCACAA | 96.4 |
|  |  | AAGCTCTGACCCTCATCACC |  |
| BolMADS55 | Bol016615 | TCGATGATGGTGTCTTGGAT | 99.5 |
|  |  | CATCTGGATTCATCGTGGAG |  |
| BolMADS56 | Bol016587 | GAACAACGAGGTGACGAAGA | 99.1 |
|  |  | ATGAGATGATCCATCGCTTG |  |
| BolMADS57 | Bol009950 | ACGATATTTGTGGCACTCCA | 102.3 |
|  |  | AGCCTCCATAAACCCATCAG |  |
| BolMADS58 | Bol009965 | GACTCCATCGTTACCGCTTT | 95.1 |
|  |  | ACCACTGAGGCAACCCTAAC |  |
| BolMADS59 | Bol030956 | AAGGAACTAACGGGTTGCAG | 97.3 |
|  |  | ATTCTGTGTTGACCCATCCA |  |
| BolMADS60 | Bol016846 | CAACAGTGCTTGATGTTCCC | 94.8 |
|  |  | CTCTCGTTTCCCTTCGACAT |  |
| BolMADS61 | Bol022880 | GCCTGAGGACAGAGAGAAGG | 99.2 |
|  |  | AAACCTCGGATGCCAGTCT |  |
| BolMADS62 | Bol002990 | CGCAACTCCCAGTCTCTACA | 97.9 |
|  |  | AGGAGCTCGTTCATTCCAGT |  |
| BolMADS63 | Bol027893 | TTCTCGAACGTGACAGAACC | 101.6 |
|  |  | ATGGGATAGTCTGCGAAAGG |  |
| BolMADS64 | Bol008049 | ATCAGTTGAGGATGCAGCAG | 98.2 |
|  |  | CCTTGCTGTTGGTGGTGTTA |  |
| BolMADS65 | Bol012873 | CACCTTTCAACATCTTTGCG | 97.2 |
|  |  | TGTAGCTCTGGTGGTTGTGC |  |
| BolMADS66 | Bol012871 | TCAAGTTCCTTGCGTTTCAG | 103.1 |
|  |  | AACGTTGCAGCTGTTGGTAG |  |
| BolMADS67 | Bol037792 | CAAGTAAGTATCTAGCGGCAATG | 95.9 |
|  |  | TTGTCGGTCGGATAACAGAT |  |
| BolMADS68 | Bol044867 | TGATCAACAACCCAAGGAGA | 98.3 |
|  |  | GATGGTTGTTCAAAGCCAGA |  |
| BolMADS69 | Bol012124 | TGGCCAACAATTTCAAGAAG | 97.3 |
|  |  | CCGTTCTTGCAACGTAGAGT |  |
| BolMADS70 | Bol010353 | CCATTTGAAGACCAGGTCAA | 99.8 |
|  |  | AAGGTTGCTTCAAAGATGGG |  |
| BolMADS71 | Bol012341 | TGATCCAAGACGCAAAGAAG | 99.9 |
|  |  | TTCCAAAGCCTTGATCCTCT |  |
| BolMADS72 | Bol012715 | CCAGCCTCCTGGTAACTTGT | 101.3 |
|  |  | CACTCATCTGCTGAGGTCGT |  |
| BolMADS73 | Bol012716 | GGAGGCTCCTGAACAATTTC | 98.8 |
|  |  | TCGAACCATTGAACATAGGC |  |
| BolMADS75 | Bol023555 | TGATCCAAGACGCAAAGAAG | 102.1 |
|  |  | GAGGATGGTTGCTCCTGATT |  |
| BolMADS76 | Bol002873 | ACCCGTTTCCTCAGAATCAG | 95.9 |
|  |  | TGGTTAGGCTGACGGTAGTG |  |
| BolMADS77 | Bol022282 | TCCTGCGTATCTCAATGGTC | 96.3 |
|  |  | AACCCATACCCTGGTTCAAA |  |
| BolMADS78 | Bol004579 | ATGCACATGATGAACCATCC | 99.6 |
|  |  | CTCGAAACCCTAGCAGAACC |  |
| BolMADS79 | Bol024509 | TTTGGATGTTGCATTTGAGC | 102.7 |
|  |  | GTTGCCATCTTCTTGGACCT |  |
| BolMADS80 | Bol002483 | TCTCCTCCCAGTTAGCAACC | 96.2 |
|  |  | CTTCCATCCAAGGAAAGCAT |  |
| BolMADS81 | Bol043965 | TGTCGAGGAGTTGAAGATGG | 98.5 |
|  |  | CCACTCGTATTCGGATGTTG |  |
| BolMADS82 | Bol036538 | CTCAATGGCTCTGGACTTGA | 99.4 |
|  |  | GAGGGAACACTTGCAACAAA |  |
